# Using the Elixhauser risk adjustment model to predict outcomes among patients hospitalized in internal medicine at a large, tertiary-care hospital in Israel

**DOI:** 10.1186/s13584-023-00580-x

**Published:** 2023-11-01

**Authors:** David E. Katz, Gideon Leibner, Yaakov Esayag, Nechama Kaufman, Shuli Brammli-Greenberg, Adam J. Rose

**Affiliations:** 1 Department of Internal Medicine, Shaare Zedek Medical Center and Faculty of Medicine, Hebrew University of Jerusalem, P.O.B. 3235, 9103102 Jerusalem, Israel; 2 Faculty of Medicine, School of Public Health, Hebrew University of Jerusalem, Jerusalem, Israel; 3 Meuhedet Health Care Services, Jerusalem, Israel; 4 Department of Quality and Patient Safety, Shaare Zedek Medical Center, Jerusalem, Israel; 5 Department of Emergency Medicine, Shaare Zedek Medical Center, Jerusalem, Israel

**Keywords:** Risk adjustment, Internal medicine, Inpatient care, Israel, Hospital mortality

## Abstract

**Background:**

In Israel, internal medicine admissions are currently reimbursed without accounting for patient complexity. This is at odds with most other developed countries and has the potential to lead to market distortions such as avoiding sicker patients. Our objective was to apply a well-known, freely available risk adjustment model, the Elixhauser model, to predict relevant outcomes among patients hospitalized on the internal medicine service of a large, Israeli tertiary-care hospital.

**Methods:**

We used data from the Shaare Zedek Medical Center, a large tertiary referral hospital in Jerusalem. The study included 55,946 hospitalizations between 01.01.2016 and 31.12.2019. We modeled four patient outcomes: in-hospital mortality, escalation of care (intensive care unit (ICU) transfer, mechanical ventilation, daytime bi-level positive pressure ventilation, or vasopressors), 30-day readmission, and length of stay (LOS). We log-transformed LOS to address right skew. As is usual with the Elixhauser model, we identified 29 comorbid conditions using international classification of diseases codes, clinical modification, version 9. We derived and validated the coefficients for these 29 variables using split-sample derivation and validation. We checked model fit using c-statistics and R^2^, and model calibration using a Hosmer–Lemeshow test.

**Results:**

The Elixhauser model achieved acceptable prediction of the three binary outcomes, with c-statistics of 0.712, 0.681, and 0.605 to predict in-hospital mortality, escalation of care, and 30-day readmission respectively. The c-statistic did not decrease in the validation set (0.707, 0.687, and 0.603, respectively), suggesting that the models are not overfitted. The model to predict log length of stay achieved an R^2^ of 0.102 in the derivation set and 0.101 in the validation set. The Hosmer–Lemeshow test did not suggest issues with model calibration.

**Conclusion:**

We demonstrated that a freely-available risk adjustment model can achieve acceptable prediction of important clinical outcomes in a dataset of patients admitted to a large, Israeli tertiary-care hospital. This model could potentially be used as a basis for differential payment by patient complexity.

## Introduction

Patients admitted to an internal medicine (IM) service in a hospital can be quite complex. By definition, these patients are ill enough to warrant hospitalization. Many have a significant list of chronic comorbidities, take multiple medications on an ongoing basis, or may have other complicating factors that are not consistently captured in structured data. For these reasons, it can be a challenge to define the complexity of patients admitted to internal medicine [1].

This difficulty can be reflected in billing practices. It is more straightforward and usually more lucrative to bill for admissions involving procedural interventions rather than for IM patients being treated for multiple non-surgical conditions. In Israel, reimbursement of hospital care varies. Inpatient procedures are paid for either per diem or through activity-based procedure-related group (PRG) arrangements, which usually pertain to the activities of surgical or procedure oriented specialties [2]. Until 2022, care provided by the IM service was reimbursed on a per-diem basis by the health plan—that is, a flat fee per patient per night. Starting in 2023, hospitals have been reimbursed through a prospective annual budget based on the previous year’s utilization. Neither of these mechanisms reflects the patient’s comorbidities or the severity of his or her clinical condition. Thus, hospitals that take care of more complex patients do not receive any extra payment.

Adjusting for complexity is important, to ensure that hospitals that treat more complicated patients receive adequate reimbursement [3]. The usual way to adjust for differential complexity between patients is to apply a risk adjustment model, also known as case mix adjustment [3]. There are many existing risk adjustment models that can be used. The idea of using them is that they account for a group of measured variables that together define how complex a patient is likely to be. These models have been shown to be very predictive of outcomes such as in-hospital mortality and length of stay, and somewhat less predictive of readmissions (which are very hard to predict) [4, 5]. Some risk adjustment models are freely available, but in fact most are proprietary. In addition to using them as a basis for differential payment to hospitals, they are also used to adjust severity in observational research studies, and by hospitals to support their operations and planning.

In most countries, some sort of risk adjustment model is applied to support differential payment for hospital care. Without such adjustment, hospitals that care for more complex patients are burdened with inadequate resources. Beginning in 1983, the US Centers for Medicare and Medicaid Services (CMS) adopted diagnosis-related groups (DRGs) as a way to group hospitalized patients by severity and expected complexity of care. The existence of DRGs was part of what enabled hospitals to reduce lengths of stay and reduce costs over time [6]. Since that time, there has been continued innovation in terms of grouping patients more precisely. For example, CMS has developed a more advanced grouping system (Medicare Severity Diagnosis Related Groups, or MS-DRG) to determine hospital reimbursement rates. Their index reflects the diversity, complexity, and severity of a patient’s illness at the time of admission. Other developed have also innovated in terms of making the DRG concept more precise over time to support differential payment by complexity [7, 8].

In Israel, there is not currently a system of case mix adjustment to support differential payment for hospital care. Here, we will demonstrate that a publicly-available system can be used successfully in an Israeli context. It is logical that we would use a publicly available case mix model rather than a proprietary one. Two popular models for estimating patient complexity are the Charlson Comorbidity Index (CCI) and the Elixhauser Comorbidity Index (ECI). The CCI is a composite score summarized by a weighted combinations of 17 comorbidities, which was originally developed to predict the risk of death among breast cancer patients [9], and has been adapted for use with administrative datasets [10, 11]. The original Elixhauser system measures 30 dichotomous variables [12], and has also been adapted for use in hospital-based case mix adjustment.

Both of these methods can be used to predict mortality, length of stay, readmission, and heath care utilization [13, 14]. Several studies have demonstrated Elixhauser’s system to be superior for predicting various outcomes [15–17]. The Elixhauser Comorbidity Index has not been used in Israel to try and define and evaluate the burden of comorbidities for patients admitted to IM departments.

The aim of this study was to apply the ECI to patients admitted to a tertiary-care center in Israel for internal medicine care, to more fully reflect the complexity and severity of the patient population treated there. We examine the ability of the model to predict the outcomes of in-hospital mortality, need for intensive care, 30-day readmission, and length of stay. We expected to show that this model could predict these outcomes here as it has elsewhere. Demonstrating the validity of this model for use in Israeli IM patients could support billing reform and help reshape the landscape in terms of reimbursement for inpatient IM services.

## Methods

### Database

We used data from the Shaare Zedek Medical Center (SZMC), a large tertiary-care referral hospital in Jerusalem that serves a varied population in terms of ethnicity and socioeconomic status. The study included 55,946 hospitalizations between 01.01.2016 and 31.12.2019. These dates were chosen to allow us to study care under usual conditions, prior to the influence that coronovirus disease 2019 (COVID-19) had on the Israeli medical system. The study unit was hospitalizations rather than patients, since some patients were admitted more than once. Another reason to focus our study on hospitalizations as the unit of analysis, rather than unique patients, is that case mix models are in part intended to support differential payment by complexity for hospitalizations, rather than for unique individuals per se.

For the purposes of this study, “internal medicine wards” were defined as the four formal IM departments (A, B, C, and D), geriatrics, cardiology, and a short stay unit. The rationale for including these additional units is that some Israeli hospitals do not have such units, and therefore the patients hospitalized at SZMC in these wards would be part of the population served by IM in a different hospital setting. In order to capture the entire spectrum of IM patients, they are included here. This study was approved by the research ethics committee of SZMC (0361-21-SZMC).

### Demographics and patient characteristics

Patient deidentified medical information was extracted from the hospital's electronic medical record. We characterized demographics, dates of hospitalization and discharge, date of birth, gender, and all wards the patient visited during the hospital stay.

### Dependent variables: patient outcomes

There were four dependent variables for this study, each of which was modeled as a separate outcome. Three are binary outcomes, and the last is a continuous outcome. The first outcome was in-hospital mortality. The second was readmission to the hospital within 30 days of the date of discharge. Because our data are limited to SZMC, readmissions to other hospitals would not be captured, but SZMC patients are primarily readmitted back to SZMC [18].

The third outcome was requiring an escalation of care beyond the regular medicine ward. Our definition of this outcome was expansive, and included any patient who spent part of their hospital stay in the intensive care unit (ICU), the intermediate care unit (IMCU i.e., a monitored setting, part of the IM ward, but with fewer patients per nurse), who received mechanical ventilation, daytime bi-level positive pressure ventilation (BiPAP), or vasopressors (i.e., medications intended to support blood pressure). Daytime BiPAP was defined as occurring between 8 AM and 8 PM. BiPAP received at night may be needed for disordered breathing during sleep (i.e., obstructive sleep apnea), but BiPAP received during the day is presumably intended as a method to prevent invasive mechanical ventilation. Vasopressors included adrenaline, dobutamine, dopamine, milrinone, noradrenaline, phenylephrine, and vasopressin. Any of these interventions (i.e., ICU, IMCU, mechanical ventilation, daytime BiPAP, or vasopressors) sufficed to show that the patient had at least some degree of critical illness and required intensive intervention. Our group has previously published about the relationship between bed location and the receipt of mechanical ventilation, daytime BiPAP, and vasopressors [19]. Some patients experienced an escalation of care, and then died during the same hospitalization. Such patients were included in both analyses.

The fourth outcome, which was continuous, was the length of stay (LOS), measured in days. Because LOS is known to be right-skewed (i.e., not normally distributed), we performed a log transformation, which did result in a normal distribution. As such, we report outcomes for the log-LOS—as is usual in many studies [20].

### Independent variables: Elixhauser index

The Elixhauser Index is a case-mix adjustment model based on patient comorbidities that predicts outcomes such as our four outcomes. Originally developed by Elixhauser and colleagues, it has since been used as a case-mix adjustment model by other investigators, such as Van Walraven and colleagues [21]. The original Elixhauser paper gives a list of diagnosis codes that are used to define 30 comorbid conditions, in the International Classification of Diseases, Version 9 (ICD-9). The codes, and the diagnoses they stand for, are in Table 1, and are taken from the original Elixhauser paper.

**Table 1:** Definitions of comorbidities

Comorbidity	ICD-9-CM codes	DRG screen: case does not have the following disorders (DRG):
1. Congestive heart failure	398.91, 402.11, 402.91, 404.11, 404.13, 404.91,404.93,428.0–428.9	Cardiac^a^
2. Cardiac arrhythmias	426.10, 426.11, 426.13, 426.2–426.53, 426.6–426.89, 427.0, 427.2, 427.31, 427.60,427.9, 785.0, V45.0, V53.3	Cardiac^a^
3. Valvular disease	093.20–093.24, 394.0–397.1, 424.0–424.91, 746.3–746.6,V42.2,V43.3	Cardiac^a^
4. Pulmonary circulation disorders	416.0–416.9, 417.9	Cardiac^a^ or COPD (88)
5. Peripheral vascular disorders	440.0–440.9, 441.2, 441.4, 441.7, 441.9, 443.1–443.9, 447.1,557.1,557.9, V43.4	Peripheral vascular (130–131)
6. Hypertension (combined) Hypertension, uncomplicated	401.1, 401.9	Hypertension (134)
Hypertension, complicated	402.10, 402.90, 404.10, 404.90, 405.11, 405.19, 405.91, 405.99	Hypertension (134) or Cardiac^a^ or Renal^a^
7. Paralysis	342.0–342.12, 342.9–344.9	Cerebrovascular (5, 14–17)
8. Other neurological disorders	331.9, 332.0, 333.4, 333.5, 334.0–335.9, 340, 341.1–341.9, 345.00–345.11, 345.40–345.51, 345.80–345.91, 348.1, 348.3, 780.3, 784.3	Nervous system (1–35)
9. Chronic pulmonary disease	490–492.8, 493.00–493.91, 494, 495.0–505, 506.4	COPD (88) or asthma (96–98)
10. Diabetes, uncomplicated^b^	250.00–250.33	Diabetes (294–295)
11. Diabetes, complicated^b^	250.40–250.73, 250.90–250.93	Diabetes (294–295)
12. Hypothyroidism	243–244.2, 244.8, 244.9	Thyroid (290) or endocrine (300–301)
13. Renal failure	403.11, 403.91, 404.12, 404.92, 585, 586, V42.0, V45.1, V56.0, V56.8	Kidney transplant (302) or renal failure/dialysis (316–317)
14. Liver disease	070.32, 070.33, 070.54, 456.0, 456.1, 456.20, 456.21 571.0, 571.2, 571.3, 571.40–571.49, 571.5, 571.6, 571.8, 571.9,572.3,572.8, V42.7	Liver^a^
15. Peptic ulcer disease excluding bleeding	531.70, 531.90, 532.70, 532.90, 533.70, 533.90,534.70,534.90, V12.71	GI hemorrhage or ulcer (174–178)
16. AIDS^b^	042–044.9	HIV (488–490)
17. Lymphoma	200.00–202.38, 202.50–203.01,203.8–203.81, 238.6, 273.3, V10.71, V10.72, V10.79	Leukemia/lymphoma^a^
18. Metastatic cancer^b^	196.0–199.1	Cancer^a^
19. Solid tumor without metastasis^b^	140.0–172.9, 174.0–175.9, 179–195.8, V10.00-V10.9	Cancer^a^
20. Rheumatoid arthritis/collagen vascular disease	701.0, 710.0–710.9, 714.0–714.9, 720.0–720.9, 725	Connective tissue (240–241)
21. Coagulopathy	2860–2869, 287.1, 287.3–287.5	Coagulation (397)
22. Obesity	278.0	Obesity procedure (288) or nutrition/metabolic (296–298)
23. Weight loss	260–263.9	Nutrition/metabolic (296–298)
24. Fluid and electrolyte disorder	276.0–276.9	Nutrition/metabolic (296–298)
25. Blood loss anemia	2800	Anemia (395–396)
26. Deficiency anemias	280.1–281.9, 285.9	Anemia (395–396)
27. Alcohol abuse	291.1, 291.2, 291.5, 291.8, 291.9, 303.90–303.93, 305.00–305.03, V113	Alcohol or drug (433–437)
28. Drug abuse	292.0, 292.82–292.89, 292.9, 304.00–304.93, 305.20–305.93	Alcohol or drug (433–437)
29. Psychoses	295.00–298.9, 299.10–299.11	Psychoses (430)
30. Depression	300.4, 301.12, 309.0, 309.1, 311	Depression (426)

ICD-9-CM, International Classification of Diseases, 9th Revision, Clinical Modification; DRG, group; COPD, chronic obstructive pulmonary disease; GI, gastrointestinal; AIDS, acquired syndrome; HIV, human immunodeficiency virus

^a^Definitions of DRG groups: Cardiac: DRGs 103–108, 110–112, 115–118, 120–127, 129, 132–133, DRGs 302–305, 315–333; Liver: DRGs 199–202, 205–208; Leukemia/lymphoma: DRGs 400–414, DRGs 10, 11, 64, 82, 172, 173, 199, 203, 239, 257–260, 274, 275, 303, 318, 319, 338, 344, 346, 347, 354, 366, 367, 406–414

^b^A hierarchy was established between the following pairs of comorbidities: If both uncomplicated and complicated diabetes are present, count only complicated diabetes. If both solid tumor without metastatic cancer are present, count only metastatic tumors

At SZMC, as in the entire Israeli health system, diagnoses are recorded using the ICD-9 system, which is compatible with this list of codes. Patients were defined as having a diagnosis if they recorded a code for it during their hospitalization. Each hospitalization was analyzed as a separate unit, so codes recorded for the same patient during a different hospitalization did not count. In using the codes, we noticed that only 6 patients had the diagnosis of iron deficiency anemia as defined by the Elixhauser model; perhaps this set of codes is not often used at SZMC, and it is likely that they are grouped instead with the “deficiency anemias” category. Because we expected such a small number to interfere with our models due to small cell counts, we deleted this condition and based our analyses on the other 29 conditions.

### Statistical analyses

We began by examining the frequency of basic information (e.g., sex, age, etc.) and the 29 comorbid conditions in the Elixhauser model. We also constructed our four outcome variables and examined their frequencies in the dataset.

When using the Elixhauser model, one is meant to derive the model coefficients anew each time. This means that the beta coefficients for each of the 29 conditions are fit to the particular dataset. In fact, we had four sets of coefficients for the model, one for each of the outcomes. We randomly divided our dataset 70/30 into derivation and validation sets. We used the derivation set to derive our model coefficients for each of the outcomes, and then forced those coefficients onto the validation set to check that model fit did not decrease (which would have indicated overfitting). For binary outcomes, which were modeled using a logistic model, model fit was characterized using the c-statistic, also known as the area under the receiver operator curve (ROC). For the continuous outcome (log LOS), which we modeled using an ordinary least squares (OLS) model, fit was characterized using R^2^.

We also characterized model calibration, which can be thought of as a measure of whether the model systematically overestimates risk in lower-risk or in higher-risk groups, and by how much. For the logistic models, we did this using a Hosmer–Lemeshow test [22]. There is not a clearly analogous test for a linear model. Analyses were performed using SPSS version 24 and R Studio version 1.3.1093.

## Results

### Dataset

The baseline characteristics for the 55,946 hospitalizations during the study period can be seen in Table 2. The unit for our analysis was hospitalization, and as such, some patients appear more than once in the data. Most admissions (86%) were via the Emergency Room, with the remainder being elective admissions or transitions between departments within the hospital. Elective admissions to the internal medicine service are very rare; therefore, the great majority of patients admitted electively were initially admitted to surgery, then transferred to internal medicine later. The median age of the patients was 74 years, and most (53%) patients were male.

**Table 2:** Baseline characteristics of hospitalizations from 2018–2019 (N = 55,946)

Male, n (%)	29,915 (53.0%)
Age, years, median (IQR)	74.0 (61.0, 84.0)
Length of stay, days, median (IQR)	4.1 (2.1, 7.8)
Death during hospitalization, n (%)	3865 (6.9%)
Emergency room / non-elective admission, n (%)	48,092 (86.0%)
Increased level of care, n (%)	7489 (13.0%)
30-day readmission, n (%)	8620 (15.0%)
Mode of transport to hospital, n (%)	
Ambulance	26,864 (48.0%)
All others	29,082 (52.0%)

*IQR* interquartile range

### Patient outcomes

Approximately 7% of the hospitalizations resulted in patient deaths, 13% involved an escalation of care, and 15% of the total admissions resulted in readmission within 30 days. The median LOS was 4.1 days (IQR 2.1, 7.8).

### Balance between derivation and validation sets

We randomly divided the sample into a derivation set (39,162 hospitalizations, or 70%) and a validation set (16,784 hospitalizations, or 30%). Table 3 shows the balance that we achieved across the derivation and validation sets regarding the prevalence of Elixhauser comorbid conditions. Hypertension and congestive heart failure were the most common comorbid conditions. The derivation and validation sets were balanced in terms of the prevalence of these comorbid conditions, and thus in the severity of their comorbid illness burden (and in their overall Elixhauser risk).

**Table 3:** Balance table

Elixhauser Group, n (%)	Overall (N = 55,946)	Derivation Set (n = 39,162)	Validation Set (n = 16,784)	P value
Congestive heart failure	14,613 (27.0)	10,181 (27.0)	4432 (27.0)	0.6
Cardiac arrhythmias	2068 (3.9)	1478 (3.9)	590 (3.7)	0.11
Valvular disease	7327 (14.0)	5128 (14.0)	2199 (14.0)	0.8
Pulmonary circulation disorder	4128 (7.7)	2875 (7.7)	1253 (7.8)	0.8
Peripheral vascular disorders	11,812 (22.0)	8214 (22.0)	3598 (22.0)	0.4
Hypertension	24,721 (46.0)	17,343 (46.0)	7378 (46.0)	0.2
Paralysis	801 (1.5)	574 (1.5)	227 (1.4)	0.3
Neurodegenerative disorder	2455 (4.6)	1706 (4.6)	749 (4.6)	0.7
Chronic pulmonary disease	6497 (12.0)	4480 (12.0)	2017 (12.0)	0.091
Diabetes, uncomplicated	6672 (12.0)	4685 (13.0)	1987 (12.0)	0.5
Diabetes, complicated	691 (1.3)	486 (1.3)	205 (1.3)	0.8
Hypothyroidism	6092 (11.0)	4,287 (11.0)	1,805 (11.0)	0.4
Renal failure	1163 (2.2)	797 (2.1)	366 (2.3)	0.3
Liver disease	1126 (2.1)	767 (2.0)	359 (2.2)	0.2
Peptic ulcer disease, no bleeding	508 (0.9)	359 (1.0)	149 (0.9)	0.7
AIDS/HIV	16 (< 0.1)	11 (< 0.1)	5 (< 0.1)	> 0.9
Lymphoma	434 (0.8)	320 (0.9)	114 (0.7)	0.087
Metastatic cancer	1269 (2.4)	900 (2.4)	369 (2.3)	0.4
Solid tumor without metastasis	4380 (8.2)	3069 (8.2)	1,311 (8.1)	0.8
RA / collagen vascular disease	1,108 (2.1)	776 (2.1)	332 (2.1)	> 0.9
Coagulopathy	1065 (2.0)	760 (2.0)	305 (1.9)	0.3
Obesity	3055 (5.7)	2,133 (5.7)	922 (5.7)	> 0.9
Weight loss	285 (0.5)	201 (0.5)	84 (0.5)	0.9
Fluid and electrolyte disorder	6874 (13.0)	4773 (13.0)	2101 (13.0)	0.4
Deficiency anemia	3749 (7.0)	2618 (7.0)	1131 (7.0)	> 0.9
Alcohol abuse	214 (0.4)	148 (0.4)	66 (0.4)	0.9
Drug abuse	71 (0.1)	54 (0.1)	17 (0.1)	0.3
Psychosis	1316 (2.5)	909 (2.4)	407 (2.5)	0.5
Depression	2354 (4.4)	1628 (4.3)	726 (4.5)	0.5

*AIDS* acquired immunodeficiency syndrome; *HIV* human immunodeficiency virus; *RA* rheumatoid arthritis

### Model performance

The multivariate regression for predicting outcomes using the Elixhauser model derivation set is displayed in Table 4. The three binary outcomes are predicted using logistic regression, while log LOS is predicted using a linear regression. Because LOS is not normally distributed, we log transformed it for the linear regression, as is customary.

**Table 4:** Multivariate regression of the derivation set (n = 37, 690)

Elixhauser Group	Mortality†	Increased LOC†	Readmission†	Log-LOS‡
Congestive heart failure	1.69*** (1.52, 1.86)	1.85*** (1.71, 1.99)	1.43*** (1.33, 1.53)	0.21*** (0.19, 0.23)
Cardiac arrhythmias	0.82 (0.65, 1.02)	1.09 (0.94, 1.26)	0.83** (0.72, 0.97)	0.02 ( − 0.02, 0.06)
Valvular disease	0.94 (0.83, 1.06)	1.58*** (1.45, 1.72)	1.04 (0.95, 1.13)	0.12*** (0.10, 0.15)
Pulmonary circulation disorder	1.10 (0.94, 1.27)	1.24*** (1.12, 1.38)	1.21***(1.09, 1.34)	0.08*** (0.04, 0.11)
Peripheral vascular disorders	1.26*** (1.15, 1.39)	1.15*** (1.07, 1.23)	1.19*** (1.11, 1.27)	0.10*** (0.08, 0.11)
Hypertension	1.05 (0.96, 1.14)	1.08** (1.01, 1.15)	1.04 (0.98, 1.10)	0.10*** (0.08, 0.11)
Paralysis	1.39** (1.03, 1.83)	1.30** (1.02, 1.62)	1.12 (0.89, 1.39)	0.31*** (0.25, 0.37)
Neurodegenerative disorder	2.13*** (1.83, 2.47)	1.96*** (1.73, 2.22)	1.19*** (1.05, 1.35)	0.27*** (0.23, 0.30)
Chronic pulmonary disease	0.89 (0.78, 1.00)	1.55*** (1.43, 1.68)	1.27*** (1.17, 1.38)	0.05*** (0.03, 0.08)
Diabetes, uncomplicated	0.94 (0.83, 1.07)	1.12** (1.02, 1.22)	0.99 (0.91, 1.08)	0.04*** (0.02, 0.06)
Diabetes, complicated	0.82 (0.55, 1.16)	0.84 (0.65, 1.08)	1.25** (1.00, 1.56)	0.15*** (0.08, 0.22)
Hypothyroidism	1.33*** (1.18, 1.48)	1.02 (0.93, 1.11)	1.13** (1.04, 1.23)	0.08*** (0.06, 0.10)
Renal failure	1.58*** (1.26, 1.95)	1.39*** (1.17, 1.65)	1.10 (0.92, 1.31)	0.10*** (0.05, 0.15)
Liver disease	1.63***(1.29, 2.04)	1.20 (0.98, 1.45)	1.14 (0.95, 1.37)	0.16*** (0.11, 0.21)
Peptic ulcer disease, no bleeding	0.90 (0.58, 1.34)	0.74 (0.53, 1.02)	1.21 (0.92, 1.57)	0.02 ( − 0.06, 0.09)
AIDS/HIV	1.38 (0.07, 7.57)	4.02** (0.95, 14.4)	0.54 (0.03, 2.94)	0.28 ( − 0.16, 0.72)
Lymphoma	1.76** (1.22, 2.48)	0.89 (0.61, 1.25)	1.38** (1.03, 1.81)	0.38*** (0.30, 0.46)
Metastatic cancer	2.45*** (2.03, 2.95)	1.00 (0.79, 1.26)	1.26** (1.05, 1.50)	0.22*** (0.17, 0.28)
Solid tumor without metastasis	2.43*** (2.15, 2.73)	0.85** (0.75, 0.96)	1.56*** (1.42, 1.72)	0.23*** (0.20, 0.26)
RA/collagen vascular disease	1.13 (0.86, 1.45)	1.12 (0.92, 1.36)	1.07 (0.88, 1.29)	0.09*** (0.03, 0.14)
Coagulopathy	2.49*** (2.04, 3.03)	1.32** (1.09, 1.60)	1.13 (0.93, 1.35)	0.25*** (0.20, 0.31)
Obesity	0.56*** (0.45, 0.69)	1.28*** (1.14, 1.44)	0.83** (0.73, 0.94)	0.00 ( − 0.03, 0.04)
Weight loss	2.16*** (1.44, 3.15)	1.13 (0.76, 1.64)	1.67** (1.20, 2.29)	0.59*** (0.49, 0.69)
Fluid and electrolyte disorder	2.52*** (2.29, 2.77)	2.18*** (2.02, 2.35)	1.33*** (1.23, 1.44)	0.22*** (0.20, 0.24)
Deficiency anemia	1.19** (1.04, 1.37)	1.06 (0.95, 1.18)	1.13** (1.02, 1.25)	0.20*** (0.17, 0.23)
Alcohol abuse	0.60 (0.25, 1.21)	1.43 (0.92, 2.15)	1.14 (0.73, 1.72)	0.14** (0.02, 0.26)
Drug abuse	0.59 (0.10, 1.96)	1.83 (0.89, 3.48)	0.77 (0.31, 1.61)	0.04 ( − 0.16, 0.24)
Psychosis	0.83 (0.62, 1.09)	1.32** (1.10, 1.58)	1.51*** (1.28, 1.77)	0.18*** (0.14, 0.23)
Depression	0.88 (0.73, 1.06)	0.73*** (0.63, 0.84)	1.04 (0.91, 1.19)	0.06** (0.02, 0.10)
C statistic	0.712	0.681	0.605	
Adjusted R2				0.102

*LOC* level of care; *LOS* lenth of stay; *AIDS* acquired immunodeficiency syndrome; *HIV* human immunodeficiency virus; *RA* rheumatoid arthritis

^*^p < 0.05, **p < 0.01, ***p < 0.001

^†^Values are odds ratios and 95% confidence intervals

^‡^Values are β coefficients and 95% confidence intervals

**Fig. 1:**
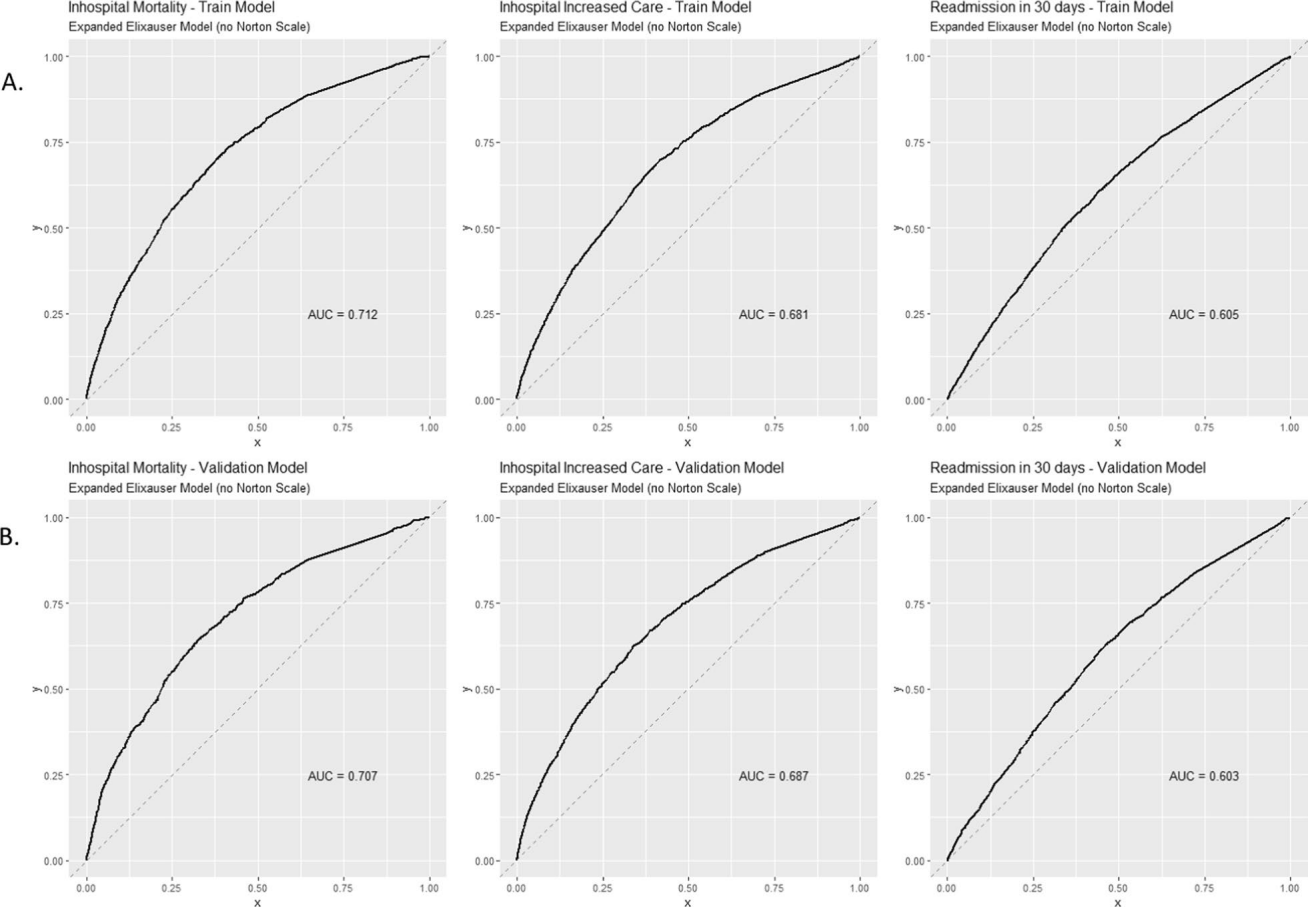
Receiver operator curves for the derivation set (column A) and the validation set (column B)

The Elixhauser model achieved acceptable prediction of the three binary outcomes, with c-statistics of 0.712, 0.681, and 0.605 to predict in-hospital mortality, escalation of care, and 30-day readmission respectively (Fig. 1). The c-statistic did not decrease in the validation set (0.707, 0.687, and 0.603, respectively), suggesting that the models are not overfitted.

The most predictive conditions for in-hospital mortality were fluid and electrolyte disorders (OR = 2.52, 95% CI 2.29, 2.77; for this and all other results discussed here *p* < 0.001, unless otherwise stated), coagulopathy (OR = 2.49, 95% CI 2.04, 3.03), and metastatic cancer (OR = 2.45, 95% CI 2.03, 2.95). The most predictive conditions for escalation of care were fluid and electrolyte disorders (OR = 2.18, 95% CI 2.02, 2.35) and other neurological disorders (OR = 1.96, 95% CI 1.73, 2.22). The acquired immunodeficiency syndrome (AIDS) was highly predictive when present (OR = 4.02, 95% CI 0.95, 14.4; *p*-value = 0.039), but impacted very few people. The most predictive conditions for 30-day readmission were weight loss (OR = 1.67, 95% CI 1.20, 2.29) solid tumor without metastasis (OR = 1.56, 95% CI 1.42, 1.72) and psychoses (OR = 1.51, 95% CI 1.28, 1.77).

The model to predict log length of stay achieved an R^2^ of 0.102 in the derivation set and 0.101 in the validation set. This means that 10% of the variation in the log length of stay was accounted for by the model variables. The most predictive conditions for length of stay were weight loss (0.59 additional days on the log scale, 95% CI 0.49, 0.69), lymphoma (0.38 days on the log scale, 95% CI 0.30, 0.46) and paralysis (0.31 days on the log scale, 95% CI 0.25, 0.37).

### Model calibration

Table 5 shows the results of the Hosmer–Lemeshow analysis for the three dichotomous variables across groups, from lowest risk (Group 1) to highest risk (Group 10). Overall, model calibration was good for all three models, with no clear systematic over- or under-prediction of risk in the low, middle, or high-risk groups.

**Table 5:** Hosmer–Lemeshow test of model calibration for in-hospital mortality (a), increased level of care (b), and 30-day readmission (c)

Risk group	Group size	Observed	Expected
*a*			
1	3811	74 (1.94%)	116.84 (3.066%)
2	1028	29 (2.82%)	38.36 (3.732%)
3	7795	203 (2.60%)	295.23 (3.787%)
4	3774	157 (4.16%)	151.78 (4.022%)
5	3768	203 (5.39%)	181.66 (4.821%)
6	3951	279 (7.06%)	236.31 (5.981%)
7	3747	312 (8.33%)	281.78 (7.520%)
8	3744	426 (11.38%)	344.13 (9.192%)
9	3769	544 (14.43%)	514.27 (13.645%)
10	2084	459 (22.02%)	525.64 (25.223%)
*b*			
1	1684	108 (6.41%)	113.81 (6.76%)
2	7795	423 (5.43%)	595.07 (7.63%)
3	3870	310 (8.01%)	313.83 (8.11%)
4	3745	361 (9.64%)	332.87 (8.89%)
5	3730	429 (11.50%)	394.17 (10.57%)
6	3900	625 (16.03%)	510.48 (13.09%)
7	3752	642 (17.11%)	579.69 (15.45%)
8	3747	745 (19.88%)	722.6 (19.28%)
9	3747	967 (25.81%)	980.4 (26.16%)
10	1501	538 (35.84%)	605.07 (40.31%)
*c*			
1	1546	150 (9.70%)	162.45 (10.51%)
2	7795	823 (0.56%)	907.7 (11.64%)
3	3748	413 (11.02%)	449.37 (11.99%)
4	3820	521 (13.64%)	504.59 (13.21%)
5	3755	573 (15.26%)	540.63 (14.40%)
6	3743	612 (16.35%)	598.06 (15.98%)
7	3755	764 (20.35%)	661.29 (17.61%)
8	3751	770 (20.53%)	740.25 (19.73%)
9	3746	878 (23.44%)	859.5 (22.94%)
10	1812	455 (25.11%)	535.17 (29.53%)

## Discussion

We examined a dataset of more than 50,000 hospitalizations from a large tertiary-care medical center in Israel during a 3-year period prior to the COVID-19 pandemic. We found that the Elixhauser model achieved acceptable prediction of in-hospital mortality, need for escalations of care, 30-day readmissions, and length of stay. These results demonstrate the feasibility of using a standard risk adjustment model, such as Elixhauser, using data from an Israeli hospital.

The IM patient admitted to the hospital has become increasingly more complex over time [23]. Comorbidities are a good predictor of in-hospital mortality [24]. In addition to in-hospital mortality, the Elixhauser approach has been used to predict length of stay, in-hospital adverse events, and is specifically tailored to be used with claims data. The Elixhauser Comorbidity Index is statistically superior to the Charlson Comorbidity Index across disease states, but to our knowledge, has not previously been applied for risk-adjusting IM patients in Israel [25, 26].

In Israel, IM hospital wards are overcrowded with patients of high complexity requiring diagnostic and treatment related services [27]. This puts extra strain on IM wards, which may not be present on more procedure-oriented services, whose reimbursement is more straightforward and more reflective of the actual amount of work involved in caring for such a patient. According to the literature, the DRG payment mechanism is the most common mechanism to pay for internal medicine care in Europe and the United States [28–30]. Under the DRG mechanism, patients are grouped by their main admission diagnosis and by severity, such pneumonia without complications, pneumonia with mild complications, and pneumonia with severe complications. These groups receive a fixed amount of reimbursement for the entire hospitalization, which in turn encourages the hospital to minimize length of stay and complications. The classification of diagnoses is based on the International Classification of Diseases (ICD) coding, usually the 10th (ICD-10) or 11th revision (ICD-11). In the DRG-system the ‘treatment nature’ varies by medical specialty. For example, in the Netherlands, there are more than 60 codes for the medical specialty ‘internal medicine’ [28].

In Israel, there are three types of reimbursement for admitted patients: per diem (e.g., medical patients), per procedure (e.g., surgical patients) or fee-for-service [31, 32]. The IM patients can be complex, but it is well-known that there are not enough ward beds in Israel to accommodate all the patients requiring an IM bed—especially in the winter, when respiratory viruses increase the number of hospitalizations to IM services [27]. In addition to these challenges, there is no appropriate system in place to reimburse hospitals according to the differential severity of patients admitted to the IM ward. It is important to emphasize that there may be two separate problems with reimbursement of internal medicine patients in Israel: unfair distribution of funds due to a failure to adjust payment for case complexity, and overall levels of reimbursement that are too low to fully cover the aggregate cost of running internal medicine departments and taking care of this population of paitents. Our contribution is to show that a case mix adjustment model can be made to work in this population. However, the application of such a model to support differential payment would only address the issue of unfair distribution of funds. If the overall level of reimbursement is too low to support hospital activities, a case mix adjustment model cannot address this part of the problem.

In this study, we have shown that the Elixhauser model can be applied and used at SZMC to capture IM patient hospitalization complexity. We established the criterion to support the validity of Elixhauser in our population, based on its ability to predict with acceptable accuracy outcomes including in-hospital mortality, escalation of care, 30-day readmission, and length of stay [33]. A weakness of our study is that it is a single center study; it would be important to repeat this exercise with other Israeli hospitals to ensure that the Elixhauser model works there as well as it did in our population; however, it would be surprising if this will not be the case.

Secondly, while the model was well calibrated across risk strata, the IM patient at SZMC is more complex than most of the other medical centers in Israel. The IM patients at SZMC are older, have more comorbidities, and a higher Charlson score [34]. These factors, combined with a local scarcity of destinations to which patients can be discharged (such as skilled nursing facilities, assisted living, and rehabilitation), lead to longer average lengths of stay at SZMC compared to other Israeli hospitals—although the median length of stay is similar to other institutions. In addition, SZMC has a relatively high percentage of 30-day readmissions compared to other Israeli hospitals [18]—which may reflect the complexity of the patient population as much as anything. Patients treated at SZMC are also older than most patients in Israel, as measured by the median age [18]. However, while SZMC may treat a population that is somewhat unusual in its high complexity, this also provides an ample population to study and apply the Elixhauser model. Lastly, we were unable to confidently identify the main reason why each patient had been admitted to the hospital. The database at SZMC does not distinguish which, if any, of the patient’s diagnosis codes represents the main reason for admission, as is often the case in databases from other locations.

To our knowledge, this is the first instance of applying the Elixhauser model to hospitalizations involving admitted IM patients in Israel. Applying a model like Elixhauser is the first step to accurately capturing the complexity of the admitted IM patient. IM patients require a holistic and individualistic approach to their treatment. Chronic medical illnesses and a long list of medications need to be addressed constantly during their very dynamic admissions, resulting in a need for work that is not well-captured by a per diem (hospital bed-day reimbursement) approach. Each illness that a patient has could be viewed more as a procedure, for which there already exists a system for reimbursement. However, this puts the burden on proper and accurate coding in order to assign the appropriate primary and secondary diagnosis, as well as severity of illness. This approach has been in place outside of Israel for many years. However, this requires properly trained staff, coders, an adequate electronic medical record, and a committed top-down government-based culture change to inpatient IM patient reimbursement. Clearly, coding cannot be perfect, and there are always inaccuracies and even incentives for intentional embellishment of codes, often called "gaming" or “up-coding” [35, 36]. Nevertheless, we would submit that an imperfect system of risk adjustment is still far preferable to none at all, because a total lack of differential payment leads to much worse market distortions than imperfect coding possibly could.

Future studies are needed to model compensation with regard to IM patient complexity in the setting of the present system in Israel, derive a new comorbidity weight on a national level, and continue to validate models such as the Elixhauser model for local use. If done properly, this could come closer to capturing the value provided by all the work performed on IM services, which is currently hard to measure.

## Conclusion

In conclusion, we used the Elixhauser model to predict major outcomes among a group of patients hospitalized on the Internal Medicine service at SZMC. Our study is a proof of concept, demonstrating that just like the Elixhauser model has worked in other settings, it can also work here. This finding can be the basis for an effort to more appropriately reimburse IM services in Israel for the work that they are actually doing.

## Data Availability

We will share our statistical code and study protocol upon request. SZMC data are available upon reasonable request and after completing a data use agreement.

## Abbreviations

SZMC: Shaare Zedek Medical Center
ICU: Intensive care unit
LOS: Length of stay
ICD: International classification of diseases
IM: Internal medicine
PRG: Procedure related group
CMS: US Centers for Medicare and Medicaid services
DRG: Diagnosis related groups
MD-DRG: Medicare severity diagnosis related groups
CCI: Charlson comorbidity index
ECI: Elixhauser comorbidity index
COVID-19: Coronovirus disease 2019
IMCU: Intermediate care unit
BiPAP: Bi-level positive pressure ventilation
ROC: Receiver operator cure
OLS: Ordinary least squares
AIDS: Acquired immunodeficiency syndrome
